# Abstracts and Gleanings

**Published:** 1881-03-20

**Authors:** 


### ABSTRACTS AND GLEANINGS

**The Opium Habit.**—Dr. C. W. Earle (Chicago Medical Review, November, 1880), writes as follows:

In these lectures on the opium habit, based on cases treated at the Washingtonian Home, and on inquiries made at fifty apothecaries' shops in Chicago, Dr. Earle states that, of 235 habitual opium-eaters, three-fourths were females, many of them prostitutes. Two-thirds were Americans by birth. The larger number were between 30 and 40 years old, of the middle class, and either were or had been married. Most began its use for the relief of pain, under direction of a physician. The form of the drug used was morphine in 120 cases, gum opium in 50, tincture of opium in 30, unknown in 27, McMunn's elixir, paregoric and Dover's powder in the remaining 8. The quantity used varied from one-third of a grain of morphia, or equivalent, to 60 grains daily. A considerable number of persons not recognized as opium-eaters used the equivalent of from one third to one grain daily, and in the confirmed cases recorded, perhaps 15 per cent. of the subjects used from one to three grains daily, 15 per cent. used three to six grains, 30 per cent. used six to ten grains, 15 per cent. used ten to fifteen grains, 15 per cent. used fifteen to twenty grains, 7 per cent. used thirty grains, and 3 per cent. used sixty grains. Only four or five persons used the hypodermic method. Some took the drug in two or three daily doses, and some a large dose at intervals of from one to three days. The effects varied very greatly, some persons being stout and rosy, some thin and sallow; but, sooner or later, there were symptoms of disordered nutrition, enervation and moral degradation. Dr. Earle regards the opium habit not as a disease, but as a vice, though he does not deny that its long continuance will produce a diseased condition; but so also, he says, will alcoholic indulgence, gluttony, tea-drinking and licentiousness. He quotes the sixth chapter of 1st Corinthians in proof that the misery resulting from the habit is continued after death, and believes it should also be punished as a vice on earth! It is hardly necessary to say that afterward, when speaking of treatment, it appears that he does not adhere to this doctrine of the necessity of the *threefold* penalty, but somewhat inconsistently does his utmost to relieve the patient. Most patients desire to abandon the habit, and it is found that it may be done, with care, with perfect safety to life. Relapses frequently occur, especially if pain or calamity supervenes. The recommencing dose will be small, but the maximum will be quickly reached. Sudden deprivation of the drug, Dr. Earle thinks dangerous, from the alarming prostration which occurs. The patient must be absolutely under control of the physician, and must be treated practically as if his word was to be doubted—though some are truthful. The last dose having been taken, he should be searched in a quiet but thorough manner, and any concealed supply removed. He will need a day and night nurse, and should never be left alone. It may take four or five days to withdraw the drug, during which time he will suffer a little. It is after the last particle is withdrawn that suffering begins, and coryza, diarrhœa, vomiting, muscular pains, and sleeplessness occur. The coryza lasts but a few days, and is treated with carbonate of ammonium, quinine and cubebs, and a snuff of morphia, bismuth and acacia. The diarrhœal discharges are dark, and quite painful. Astringents and acetate of lead are to be used. The vomiting comes with the diarrhœa, and is controlled with carbolic acid, bismuth and ice. The muscular pains are the most troublesome symptom, unless it be the feeling of depression or letting down, which early occurs. The hot bath is useful, and any anodyne not containing opium. Iron, bark and strychnia are used early and throughout the treatment. Bromide of sodium has been of use. The fluid extract of cocoa has been found of great use in the late cases in which it has been tried, if the stomach will retain full doses, and enables the morphia to be withdrawn more promptly. In one case, however, there was reason to fear the formation of a cocoa habit. Sleep usually comes after five or six nights, but in nervous patients chloral or some hypnotic should be given, with caution, lest a habit should be formed. The symptoms are not as severe where the drug has been used hypodermically. A patient will resort to any subterfuge to get the drug, and until several days or weeks have been passed without it, his moral sense is in abeyance, and he cannot be trusted. The various "opium antidotes" in the market are composed of some form of opium, belladonna, canabis indica, chloral, etc. Dr. Earle believes that physicians' prescriptions containing opium, chloral or alcohol should bear a printed request to the apothecary not to renew them without direction, and that the greatest caution should be used in prescribing and continuing such drugs. A patient should never be taught to use a hypodermic syringe.

Dr. J. D. Irwin, in St. Louis Clinical Record, reports the following case of opium habit treated with cocoa:

In this case of opium habit treated with cocoa, a lady, having begun to use morphia for the relief of pain, at last reached the amount of sixteen grains daily. Thirty hours after having relinquished it, she was found in great agony, excitement and restlessness. Bromide of potassium and hydrate of chloral were used in large doses through the night, to allay excitement and produce sleep. The next morning she was very weak and restless, scarcely able to speak, troubled with vomiting, and with a pulse of one hundred and fifteen. The fluid extract of cocoa was given in tablespoonful doses. The first dose produced little effect. The second was followed by a wonderful change; the pulse fell to 85, her face was flushed, the vomiting ceased, her countenance was lively, she talked and laughed quite freely, and in the afternoon was able to sit in a chair. She slept about half the next night, and woke quite lively and refreshed, with a pulse of 75. She enjoyed and digested her breakfast. She continued to improve, in two days took a long drive, and the next day left the city with an eight-ounce bottle of the cocoa, which she took in smaller and smaller doses, and then, relinquishing it, enjoyed good health without the aid of morphia.—*New York Medical Journal.*

**Minor Anæsthetics.**—We close our notices of the several anæsthetics with a consideration of the minor ones.

*Bichloride of methylene* is one of these. It hasn't much rank—is chiefly celebrated as being the anæsthetic under which Spencer Wells has done a number of his ovariotomies—two hundred and eighty indeed. The anæsthetic committee speak of it as bichloride of methylene *so called*, as its boiling point is not definite, and it is evidently a mixture. Dr. Kappeler charges it with nine deaths, and his conclusions are "that experience shows it to be as dangerous, if not more dangerous than chloroform." The experiments of the anæsthetic committee confirm this view, its depressing action on the heart being marked.

*Dichloride of ethedene* is the result of the study of the anæsthetic committee for some agent less dangerous than chloroform. They believe that they have found it in dichloride of ethedene—"an isomeride of ethene dichloride produced from aldehyde." Experiment with lower animals showed it a perfect agent, producing prolonged anæsthesia without cardiac depression. It reduces blood-pressure, but by regular gradation, and not suddenly, as in the case of chloroform. Vomiting is sometimes produced by it. The conclusion of the committee is that although ethedene is not free from danger, it is in a very high degree safer than chloroform; that it is less exciting, more agreeable than, and as rapid in action as chloroform, and that "a very strong case is made out for an extensive trial of it." The clinical account is not extensive, but so far favorable. Dr. Kappeler records, however, that "unfortunately the occurrence of a death from the article in Berlin destroys early the hopes which had been cherished of it, and will not permit of a more extended trial." Dr. Reeve rightly enough regrets that no account of this death is given, that we might judge how much so spotless an anæsthetic was at fault.

*Bromide of ethyl* is the remaining anæsthetic considered by Dr. Reeve. We are indebted for its introduction in America to Dr. Turnbull, who cliams originality in its use upon man, and his able coadjutor, Dr. Levis. Dr. Nunnelly, of Leeds, experimented on animals with it as early as 1849, and his experience with it as an anæsthetic with patients was the subject of a paper read by him before the British Association in 1865. Dr. Levis, upon the basis of seventy-one administrations, declares he has "used it under the most varied circumstances which could be required to test the merits of an anæsthetic, . . . . and in the most abnormal conditions of debility and shock of injury; in capital operations, through protracted periods of administration, in patients from early infancy to extreme old age;" and declares his conviction that it is practically the best anæsthetic known to the profession." These were brave words of Dr. Levis, and he had not written them very long before a death at his clinic taught him that the prophecy of anæsthesia must be built not on tens, but on tens of thousands observations. Another death on the bromide of ethyl, recorded by Dr. Sims, also hastened to dispel a fancied security in the new agent.

The bromide of ethyl is put foward with the usual claims for preference—safety (of which we have seen), quickness and pleasantness of action. These points are not thoroughly proved. It may be remembered that the *Louisville Medical News* was one of the first journals to record clinical experience (in the person of one of the editors) with the new agent. The account was not strikingly in its favor.

As we cast about for closing words which will sum up the question of anæsthesia, those which are used by Dr. Reeve are better than any we can put together, and we give them entire:

Our task has been executed during a period of greatly renewed interest in and study of anæsthetics. In regard to some the ink had scarcely dried before new facts were presented for consideration or statements had to be modified. This awakened interest in the subject, and this rapid investigation of new agents attests at once the great importance of the subject and the earnest desire of the profession for a pleasant anæsthetic safer than any we now have. Such an one is not yet found. Every one must deeply regret that a death should have occurred to shake confidence in an article which promised a realization of our hopes. Yet when we reflect on the number of successful inhalations necessary to prove that a new anæsthetic is better than those already tried, the difficulty of the problem is appreciated. The new candidate must have been used thousands of times before it can rival chloroform or the mixed vapors, and tens of thousands before it can be compared with either in regard to safety. Were this fact kept in view there would be fewer journal articles and less dogmatism on this class of remedies. Is it probable that a perfectly safe anæsthetic will ever be at our command? The time necessary for the proving of new agents makes this very improbable during the lifetime of those who saw the advent of ether and chloroform, and the retrospect we have made is not favorable to an affirmative answer. Disaster has followed the use of all, and the facts now will sustain Velpeau's statement as well as when he made it, and still justify Erichsen's words: "With every possible care it appears certain that the inhalation of any anæsthetic agent is in some cases almost inevitably fatal." The inference is that what has been so universally true in the past will be true in the future, however sad this may be to the philanthropist or to the enthusiastic surgeon. Unfortunately physiology teachss the same lesson: a state of artificial anæsthesia is a state upon the borders of death.

The great practical lesson of all facts and all theory is that in the use of anæsthetics no precaution can be superfluous, no care too minute, and no watchfulness too great.

**Recent Studies on the Nature of Malaria.**—*Le Journal d'Hygiene* was the first scientific organ which made known in France the remarkable work undertaken in Italy by Professors Edwin Klebs and Tommasi Crudeli—work which has led these *savants* to a discovery of the highest importance, viz.: To know the specific agent of malaria. The following are the results obtained by these new observers:

1st. In all the malarial country of the Roman Field they have found the *bacillus malariae* already developed (Cuboni—and, by artificial culture it can be produced in great quantity). It has not been met with in the country in the healthy localities of Lombardy.—(Cuboni.)

2d. This same *bacillus* is accumulated sometimes in such considerable quantities in the strata of air which lie over the malarious districts during the hot days of summer that, to gather it, special apparatus is useless. It is found in abundance in the sweat of the forehead and hands.—(Cuboni.)

3d. During the height of the fever, sporules of the *bacillus malariae* are constantly present.

(*a*) They are also found in the blood of hares, which have been subjected to the malarial infection.—(Ceci.)

(*b*) In the blood drawn from the veins of men similarly tainted.—(Marchiafava, Perrancito, Ferraresi.)

(*c*) In the blood taken from the spleen of the same invalids (after a process conceived by Dr. Sciammanna).

(*d*) By the examination of this blood, has been obtained the *bacillus*, perfectly developed, and presenting the same forms already described by Klebs and Tammari.—(Crudeli, Ceci, Cuboni, Ferraiesi.)

(*e*) The same has also been obtained by the examination of the spleen of persons dead of pernicious fever. Examinations made of the spleen of those dying of other maladies in non-malarious regions give negative results.

4th. If the blood drawn from the veins of invalids, tainted with marsh fever, is injected in the subcutaneous tissue of dogs, it produces in these animals the same disease.—(Marchiafava, Ferraresi, Valenti, Sciammanna, Piricilli.)

5th. In all cases where the blood of those tainted with malarial fever has been drawn from the veins during the inception of the fever, this blood usually contains the fully developed *bacillus* in considerable quantities. In the height of the fever, on the contrary, as we have already said, the *bacillus* disappears to give place to its sporules.—(Marchiafava, Ferraresi.)

The invariability of this last result, analogous to the facts observed in the study of *spirillum*, which causes typhus, is of considerable importance in the question which now occupies us. First of all, it gives us the explanation of the differences of the results obtained during the summer of 1879 by Prof. Marchiava, in the series of examinations made of the blood of five persons dead of pernicious fever—the examinations being made immediately after death. In three of these the blood in the veins and heart contained a great number of fully developed *bacilli*, while it was impossible to find in the blood of the other two a single *bacillus*, but only a remarkable quantity of spores. Facts newly discovered at Rome lead us to believe that the first three died during the period of invasion; the other two, on the contrary, must have perished during the crisis of the fever. Experiments on animals have demonstrated that the chosen seats for the parasite which produces malarial fever, are the spleen and the medulla of the bones, organs which invariably are the seat of the gravest pathological changes in persons who have succumbed to this affection. It is probable that the production of the new generations of the parasite in these organs varies in rapidity according to individual disposition, and perhaps also according to the quality of the land whence the parasite comes; this will explain the great variety which is observed in the duration of the intermission in marsh fever. Probably the occasion of fever is produced only at the moment when the emission of the parasites, coming principally from the spleen, comes to a point similar to the stage of those found in quantities in the blood. Perhaps, also, the cold of the febrile invasion is due to the irritation of the vaso-motor nerves following the presence of this army of invaders in the circulatory system. These organisms find in the blood the most favorable conditions, for accelerating their evolution, viz. : heightened temperature and oxygen stored up in the red globules. It is not astonishing that their disorganization should be already complete at the height of the fever. On the other hand, the great number of reactions undergone in the blood and tissues, and the resulting assimilations and excretions, sufficiently explain the development of the febrile heat.—*Virginia Medical Monthly*.

**An Interesting Case from Practice**.—Dr. W. A. Fannin, in Monthly Review of Medicine and Pharmacy, says:

Mrs. B. called at my office at 12:30 p. m., August 3d, 1879, bringing with her a child thirteen months old. The child was breathing heavily, and partially unconscious, and had had numerous passages, green in color, with white particles in suspension. Pulse 160, temperature 107½°. Examination of lungs: Right side slightly dull on percussion over lower lobe; auscultation; coarse mucous rales over both lungs. I ordered

Potass. nitrat.,............ ................................ gr. j.
Ex. hyoscyami,..................... .................... gr. 1-12.
Tr. aconiti rad.,.................. ......................... gtt. ss.

to be given every two hours.

5 p. m.—Pulse 160. Temperature 107½°. Respiration shallow and abdominal; eyelids partially closed; eyeballs turned up and in divergent strabismus, and in a state of tremor. Patient had a movement every five minutes at least, and of same character. I told the mother the child would die in half an hour, and proposed a cold bath, by which the temperature fell to 102°; same internal treatment.

9 p. m.—Child had one passage in the interval of time; rested well and recognized the parents. Temperature 106½°. I gave another bath; while in the bath the child fell asleep. On taking it out of the bath respiration ceased, which I easily re-established. Temperature 98°; breathing regular and the child asleep; bowels bloated.

August 4th.—Child passed a quiet night. No movement of fæces, but passes large quantities of wind. Temperature 103°. Coughs a little. I ordered cold cloths to the abdomen and head; same internal treatment.

1 p. m.—Temperature 101°. No movement of the bowels; nurses well when awakened. I bandaged the abdomen to control the flatus.

9 p. m.—Temperature 100°. No movement. I administered ℥ij castor oil, and ordered

Potass. citrat.,

" nitrat.

Ammon. carb.,...................................... aa gr. xxxij.

Hydrargyri chlor. corros.,........................ gr. ⅛

Aq.................................................. ℥ iv.

Sig. Teaspoonful every two hours, and advised an excursion on the water for the next day.

Aug. 5th.—Been on the water; the oil operated twice; flatus gone; stool of right color, and the child has been bright all day.

Aug. 6th.—Been on the water again; had one movement, normal in color, etc. Same internal treatment.

Aug. 7th.—Temperature 99°. Stools normal; child discharged cured.

This case shows that even extreme cases should not be given up as hopeless. I have seen comparatively hopeless cases likewise benefitted by the cold bath, and I never hesitate to administer it when the child has a temperature over 103°. That the cure was due in this case to the bath cannot be doubted, as the internal treatment was directed to the fever only.

**The Use of Anæsthetics in Labor.**—Few surgeons hesitate to use anæsthetics in almost all surgical operations at the present day, whether they be great or small, and with but little or no risk if a pure drug is judiciously used. We must admit, if we are honest with ourselves, from the foregoing, that in the majority of surgical operations the death tendencies are much more evident than they are in the majority of obstetrical cases.

Death, following a surgical operation, is generally the result of shock, anemia of the brain or general debility, all of which we are compelled to admit anæsthesia very greatly favors.

Death, in the parturient condition, as a rule, is either from hemorrhage, over-exertion, or consequent reaction, plethora, congestion of the brain or convulsions, all of which, except the former, find a ready and reliable antagonist in our anæsthetics—and the danger of the former are not in the least degree increased.

These facts naturally press us to the legitimate conclusion that the parturient woman is physiologically in a safer condition for the use of an anæsthetic than the surgical patient; while no surgeon questions the propriety of giving chloroform in almost any case, whether it be a capital or minor operation.

Then, why should we not use anæsthetics in labor as well as in our ordinary surgical operations when they are not contra-indicated by her condition physiologically, but on the contrary act favorably?

The past experience of our best accoucheurs who have been using anæsthetics for a score of years, almost with one accord testify to its happy results upon the mother's health, whilst in all cases it has soothed their suffering and saved them from the excruciating expulsive sive pains of labor. By reference to my private notes on obstetrical prac-practice, I find that one ot the most careful and guarded of instructors in the University of Pennsylvania, who is always careful not to venture an opinion before he is satisfied with its orthodoxy, Prof. R. A. F. Penrose, in a private conversation with him some years ago, gave it as his opinion then, that the careful and judicious use of anæsthetics in the later stages of labor, and in hard labor, and especially in dystocia, was certainly one of the modern God-sends to the suffering female; but also added that great care should be used to guard against hemorrhage, and especially in those persons who were predisposed to it. For, said the professor, its application in this department of the medical science is comparatively recent and demands the closest scrutiny on the part of the accoucheur.

In a private letter from my esteemed and worthy friend, Prof. John J. Reese, of the University of Pennsylvania, who first instructed me in this branch of our healing art, and who has had the experience a score of years affords in this particular direction, says: "I have no reason whatever to change my views as regards the efficiency of anæsthetics in labor; I think they are not called for in ordinary mild cases, unless the patient is very nervous and timid. But I never fail to use them in hard labors, and always in instrumental ones." Cazeaux says that "when properly administered, and in moderate doses, anæsthetics do not interfere with the regular course of the uterine contractions." Instead of the wild excitement, horrified with the possibilities of impending dissolution, by a proper and careful etherization of the patient, she passes into a quiet and peaceful slumber while the laboring uterus continues its course uninterrupted. The soft parts are aided in their relaxation, while a strong barrier is placed against that dreaded and oftentimes fatal calamity, puerperal convulsions. Thus the labor proceeds quietly and peacefully to a happy end, and the wild shrieks and sobs of despair so common on these occasions are silenced by a peaceful rest in the arms of morpheus.

Having satisfied ourselves that the results from the use of anæsthetics in labor are beneficial rather than injurious to the mother, we will glance at its effects on the fœtus. Cazeaux says, that "whatever difference of opinion may still remain respecting the influence of chloroform upon the health of the mother, no one doubts its entire innocence as regards the fœtus. In the immense majority of cases the new-born child presents its usual appearances; its cries are neither weaker nor heard less promptly, nor does its viability appear to be in any way injured."

We have not, and will not, lengthen this by proving our statements with a detail of cases which can be found by those who wish to investigate the subject still further in almost any obstetrical journal or standard work on obstetrics. We have simply given you a summary of the conclusions we have arrived at from our own experience in the use of anæsthetics in labor, backed by that of standard authorities whose experience of a score of years has led them to confirm their use.

Thus far we have said nothing about the use of anæsthetics in dystocia, in which condition they are absolutely invaluable, not only for the relief of pain from any operation which it may be necessary to subject the patient to, but by rendering the patient motionless, and thus facilitating any manipulation which may become necessary.

The objections to the use of anæsthetics in labor, lest you should be obliged to give other remedies, is only a popular bug-bear and without foundation; for in obstetrical practice it is seldom if ever necessary, except in some operation, to push anæsthesia to perfect unconsciousness, but simply to the relief of pain, and in that condition there is seldom any trouble in getting patients to take almost any nostrum you may desire.

It is scarcely ever necessary to use an anæsthetic before the last stages of labor, although it is not entirely objectionable.

Prof. John J. Reese says, in the same letter referred to before, that as a rule in his private practice he does "not administer them until the head is well engaged and the labor pains become decided. I do not keep the patient all the time fully etherized, but let her inhale a fresh dose just as the pains come on, and very generally she is unconscious of the last pain, and remains so for a few minutes after the expulsion of the child.

In my own practice my rule is not to commence the use of the anæsthetic before the os is dilated to, at least, the size of a common nickel, before which the woman will bear her pain with but little complaint. I have found that the danger of delay in labor is much more liable to occur from the use of an anæsthetic too early in labor than by the too free use of it after labor has advanced.

It may be laid down as a rule before commencing the use of an anæsthetic, to give a dose or two of the fluid extract of ergot which will not only facilitate the uterine contractions, but act as a safeguard against any tendency to hemorrhage. Of late I have generally used quinia in from three to five grain doses or the two combined, and have always obtained the most happy results from their use.

A diversity of opinion still exists as to the best and safest anæsthetic to use. Cazeaux advises the use of chloroform; Prof. Reese uses pure washed ether, six parts, to pure chloroform one part, to be mixed just before using; Dr. Barr of Philadelphia uses ether three parts, chloroform one part, and alcohol, two parts. The formula which I have been using is

℞ Chloroform (Squibb's) ........................................... ℥ ii

Aeth. sulph. (Squibb's)............................................ ℥ ii

Alcohol absolute (Squibb's)......................................... ℥ j from which I have always had the most desirable results.

We trust the few suggestions we have given you may receive a fair and impartial trial, especially by those members of our profession who are as yet strangers to the use of anæsthetics in obstetrics; and may they seek early to use the elements which God Almighty has placed at their command, and set them as guardians over, with which they can calm the troubled sea of pain and agony, and say "Peace be still."

Before closing let me ask you, Is it fair, just and honorable for you to favor the stalwart man with this boon of relief for a trifling operation, or endanger without hesitation the feeble patient who comes to your office with one foot in the grave and the other on its very brink, and yet deny our mothers, wives, sisters, daughters and the female world at large, who year after year travail in pain and anguish together, and yet who are physiologically well, and who run less danger from its use than the former? Just think of it for a moment, and ask yourself the question, Is it not more from prejudice than reason?

**Syphilitic Ulceration of Velum accompanied with a Large Perforation.**—The patient when first examined had all the evidences of tertiary syphilis in the buccal cavity. The entire mucous membrane covering the fauces was greatly congested, the inflammatory redness being of a dusky color, a characteristic peculiar to syphilitic cases, and one that will often enable the expert examiner to instantly diagnose the disease correctly. On the right side of the soft palate, midway between the base of the uvula and the attachment of the velum to the side of the throat, an opening large enough to allow of the passage of an ordinary lead-pencil was distinctly visible. The entire circumference of the above orifice was in an ulcerated condition, and the tissues for at least a line beyond looked as if they were about to become involved in the destructive process. The cause of all this trouble was so apparent that it was hardly necessary to ascertain the patient's preliminary history. In response to the usual questions, the patient replied "that he had contracted chancre twenty years ago, and that he had suffered from pains in the bones for a long time."

The stereotyped course of treatment in a case such as the one portrayed above would be to freely cauterize the edges of the perforation with the stick of nitrate of silver, and to administer internally the antisyphilitics, mercury and potash, either singly or combined. Now, instead of pursuing this plan, I, following an idea which had been applied by me to several other forms of syphilitic throat troubles, made no local application whatsoever, but simply prescribed the iodide of potash in large doses frequently repeated. The result was most gratifying, for at every attendance of the patient the perforation was smaller, until finally it disappeared altogether, the entire treatment occupying but ten days. There were several physicians who were attending the hospital as students during the treatment of the last-mentioned patient, and who followed up the case in all its stages with great curiosity. They, one and all, expressed themselves as greatly surprised at the wonderful result produced.

With reference to the throat, there is no doubt in my mind regarding the powers of the system to restore lost tissues, providing the destructive process has not gone too far, and the proper plan of treatment be pursued. I have in several marked instances seen a partially destroyed cord, which had superinduced a great amount of hoarseness, return to its normal condition with a complete restoration of the vocal powers. I have also noticed the same thing repeatedly with reference to some of the adjoining organs.—*Medical Record.*

**Iodine as a Specific in Croupous Pneumonia.**—The treatment of croupous pneumonia has some time since lost its former active character; since we could but recognize that the course of the disease could not be influenced by any of the present plans of medication. A startling statement now appears by Dr. F. Schwarz, in the Deutsche Medicinische Wochenschrift, (No. 2, 1881,) who claims that the disease may be abated by iodine or iodide of potassium. He quotes statistics in the first place from the most reliable German sources, according to which the crisis occurred in 0.6 per cent. during the second day, in 4.7 per cent. during the third, in 7.4 per cent. during the fourth, in 15.9 per cent. during the fifth, in 13.6 per cent. during the sixth, in 22.8 per cent. during the seventh, in 11.8 per cent. during the ninth day. These statistics refer to 933 cases of croupous pneumonia treated expectantly. In opposition to these figures Schwarz gives his results as the proof of the efficacy of his treatment. He has had altogether 98 cases, in ten of which the abortive treatment succeeded. This treatment is successful only when begun as soon as the disease starts. If instituted later it seems to be powerless. The inferenee to which the author leads us is that he saw none but the ten cases quoted at a sufficiently early period, but he does not state this point in so many words. By reproducing the temperature curves he proves that the aborted cases commenced in the usual characteristic manner. In all of these the crisis was completed during the second day. The defervecence took from 6 to 18 hours, on an average about 12 hours. The quantities given were very small, one sixth of a drop of tincture of iodine, or about one grain of iodide being taken every hour. After the fever had ceased the local symptoms began to recede rapidly.—*Chicago Medical Review.*

**The Prevalence of Leprosy in the United States.**—At the meeting of the Academy of Medicine, on January 20th, Dr. H. G. Piffard read a paper on leprosy. In this paper and in the subsequent discussion, some facts of much interest, and perhaps of great importance, were brought out. From the statistics collected by the Dermatological Society, it appears that there are between fifty and a hundred lepers in the United States at present. Moreover, an examinatlon of the tables show that this number has been constantly increasing every year. In view of these facts the question of the contagiousness of leprosy is a most important one, and it was discussed very carefully by the reader of the paper and other gentlemen present at the meeting referred to. Dr. Piffard was inclined to believe that, though not contagious in the ordinary sense of the word, it might be so through the medium of the blood or secretions, as in the case of syphilis. Furthermore, it was a well-established fact, that when leprosy had once gained a foothold in any community it was very sure to spread in some way. A marked illustration of this was to be seen in the Sandwich Islands. Forty years ago there was no leprosy there; now one-tenth of the inhabitants are lepers. Honolulu, a place once entirely free from leprosy, now has two hundred and fifty cases of the loathsome disease.—*Medical Record.*

**Report on Yellow Fever in the U. S. S. Plymouth.**—It will be remembered by most of our readers that yellow fever broke out in the steamer Plymouth under very strange circumstances The steamer had been on duty in the tropics, and several cases of yellow fever had occurred on her. She then rerturned to Boston in the winter. Here she was thoroughly disinfected by the best known methods, and further, every part of the vessel opened and exposed for a long time to the frost of a very severe winter. But, in spite of all this, as the ship neared the tropics, before she had come in sight of any land, several cases of yellow fever broke out on ship board. A commission was now appointed by the Navy Department to investigate the causes of this phenomenon. The entire report is worthy of study by all interested in the destruction of the elements of the disease, or in preventing their ravages. It appears that in this case the timbers of the ship were so constructed as to prevent the disinfecting agents from reaching some of the worst decaying spots. It is clear that the germs of disease remained unharmed in the rotten timber, in spite of the intense cold and disinfectants. The report further says that "the use of carbolic acid, chlorine and its compounds, sulphate of iron, nitrate of lead, and chemical disinfectants generally, are however effectual in destroying odors, of very doubtful efficacy in destroying the germs of disease." It excepts sulphurous acid gas. This seems to have a special power in the destruction of animal or vegetable life. Besides, the filling of the vessel with steam at a temperature of 250° Fahr. seems to be a convenient and effective mode of destroying the elements of disease.—*Detroit Lancet.*

**Dermic Dressing in Variola.**—Dr. A. S. Hudson says, in Med. and Surg. Reporter: Pulverized talc, or steatite, is a most elegant dressing for moist skin affections of both infants and adults. For ten years it has, in my hands, proved a useful medicament and choice vehicle, topically employed. During the last three months some thirty cases of small pox have come under the writer's observation, in which steatite has answered a most desirable object. Sweet oil and glycerine, mixed in equal parts, is applied for two or three days to the heated skin and forming pustules. As soon as the pustules are matured, or turn yellow, the steatite is applied over the face, neck and hands, mixed in water, in the form of paste or whitewash; repeated as often as it falls off or is rubbed off.

It is an energetic absorbent, a deodorizer, overcoming that evil smell peculiar to variola. It controls the "itching burning" of the surface. It prevents the second formation of crust over the pustules, and thereby renders the pits in the skin less distinct. To the touch it is soft as velvet and as adherent as bronze. For the above named needs it seems to be the Geodine of earths.—*Med. and Surg. Reporter.*

**Fuchsine in Bright's Disease.**—Prof. Renzi says, in *Pharm. Cent.*, that fuchsine (red aniline free from arsenic), may be used to prevent the formation of albumen in the urine. He administers it in the form of pills of 2½ centigrammes (¾ grain) each, beginning with two pills, 0·05 (4-5 grain), and increasing the dose to 0·25 (4 grains) in twenty-four hours. The urine becomes red and continues so during the treatment, and the appearance of the color is an indication of the efficacy of the remedy; if it does not appear, it is prevented by some organic hindrance which renders the remedy in such cases useless. Fuchsine will also cause the mucus, which is often found in the urine during this disease, to disappear.—*Drug. Cir.*

**Treatment of Asthma with the Induced Current.**—Dr. I. Burney Yeo relates, in the Lancrt, his experience at Neuenahr, where he saw the induced current used in the treatment of asthma. It sometimes acted like magic, curing the cases completely in a week or two. The electrodes are applied usually on each side of the neck, about an inch below the angle of the jaw. The current must be of good strength, so that the patient can feel the stream go across the larynx and soft palate. In bad cases it should be applied twice a day, from fifteen to thirty minutes each sitting. Dr. Max Schaeffer, who first advocated this treatment, found that the constant current never did any good.—*New York Medical Record.*

**On Cholera Infantum.**—A writer in the Medical and Surgical Reporter, thus gives his experience in the treatment of Cholera Infantum:

At the commencement of my professional career, twelve years ago, I lost, in rapid succession, several cases of cholera infantum treated according to the methods indicated in the standard medical works of that time. Soon after, I saw, in some medical work published in New York city, the histories of ten or twelve cases treated successfully with small doses of bromide of potassium. I immediately began its use, and in connection with subnitrate of bismuth and rigid dietary regulations nearly every case since, coming under my care, has terminated in recovery. The subnitrate is given every three hours, in doses of five to fifteen grains, and the following formula of the bromide is used:

℞ Potassi bromidi........................................ gr. xvj.

Aquæ destillatæ. unciam.

Sig. Coch. min. quaque hora sinnen deun.

If the child has not been weaned the diet of the mother is carefully regulated. She is restricted to toasted bread, a little milk or egg, with tea and coffee, if the latter are habitual.

The flour of which the bread is made is inspected, that it may be known to be good. The child is allowed to nurse only once in three hours, and the amount is diminished. Idiosyncrasies, if any, of the child are detected. Thus in one case a mouthful of chicken taken by the mother, in another two teaspoonfuls of milk from a certain cow, used in tea, acted as a cathartic upon their respective infants. The mother is directed to obtain at least eight hours' sleep daily, and to effect this the child, during that time, is committed to the care of a nurse. Of course the mother is roused to nourish the child, but this being performed she is again permitted to sleep. If the infant has been weaned it is confined to cow's milk diluted with rice-water, varying the proportions according to circumstances.

The milk, if possible, is given while it yet contains the animal heat, and, of course, is milked from the cow as required. The cow now occupying the office of mother must be in good health, should be in a stable perfectly clean and ventilated, furnished with abundance of food and pure water, and in no way irritated. Rigid adherence to the above plan, modified more or less by circumstances, has, as above stated, been uniformly successful for the past ten years, and being so long continued cannot be attributed to good fortune, neither to climate, as it has been equally successful in New England, Virginia and Kansas.—*Monthly Review.*

**Pilocarpin in Diphtheria.**—The effect of pilocarpin in diphtheria is, according to various German writers, most admirable. Dr. Guttman says that, given internally a free salivary discharge is established, by which the diphtheritic membranes are softened and dissolved, the inflammatory phenomena rapidly lessen and disappear; and the condition of the patient is speedily improved. Out of sixty-six cases, many of them severe, he *did not lose one*, and they all were cured in from twenty-four hours to three days!—*Med. and Surg. Rep.*

